# Selenium-enriched yeast modulates the metal bioaccumulation, oxidant status, and inflammation in copper-stressed broiler chickens

**DOI:** 10.3389/fphar.2022.1026199

**Published:** 2022-10-14

**Authors:** Ola A. Habotta, Xiaoyan Wang, Hamzah Othman, Abdulrahman A. Aljali, Mahmoud Gewaily, Mahmoud Dawood, Asmaa Khafaga, Amr I. Zaineldin, Rajeev K. Singla, Bairong Shen, Heba I. Ghamry, Eman Elhussieny, Amany El-Mleeh, Samah F. Ibrahim, Ahmed Abdeen

**Affiliations:** ^1^ Department of Forensic Medicine and Toxicology, Faculty of Veterinary Medicine, Mansoura University, Mansoura, Egypt; ^2^ Department of Pathology, Clinical Medical College and the First Affiliated Hospital of Chengdu Medical College, Chengdu, China; ^3^ Department of Pathology and Clinical Pathology, Faculty of Veterinary Medicine, Omar Al-Mukhtar University, Al-Bayda, Libya; ^4^ Department of Pharmacology, Toxicology and Forensic Medicine, Faculty of Veterinary Medicine, Omar Al-Mukhtar University, Al-Bayda, Libya; ^5^ Department of Anatomy and Embryology, Faculty of Veterinary Medicine, Kafrelsheikh University, Kafrelsheikh, Egypt; ^6^ Department of Animal Production, Faculty of Agriculture, Kafrelsheikh University, Kafrelsheikh, Egypt; ^7^ The Centre for Applied Research on the Environment and Sustainability, The American University in Cairo, Cairo, Egypt; ^8^ Department of Pathology, Faculty of Veterinary Medicine, Alexandria University, Edfina, Egypt; ^9^ Animal Health Research Institute (AHRI-DOKI), Agriculture Research Center, Kafrelsheikh, Egypt; ^10^ Institutes for Systems Genetics, Frontiers Science Center for Disease-Related Molecular Network, West China Hospital, Sichuan University, Chengdu, China; ^11^ School of Pharmaceutical Sciences, Lovely Professional University, Punjab, India; ^12^ Department of Home Economics, College of Home Economics, King Khalid University, Abha, Saudi Arabia; ^13^ Department of Veterinary Pharmacology, Faculty of Veterinary Medicine, University of Sadat City, Sadat City, Egypt; ^14^ Department of Veterinary Pharmacology, Faculty of Veterinary Medicine, Menoufia University, Shebin Elkoum, Egypt; ^15^ Clinical Sciences Department, College of Medicine, Princess Nourah bint Abdulrahman University, Riyadh, Saudi Arabia; ^16^ Department of Forensic Medicine and Toxicology, Faculty of Veterinary Medicine, Benha University, Toukh, Egypt; ^17^ Center of Excellence in Screening Environmental Contaminants (CESEC), Benha University, Toukh, Egypt

**Keywords:** copper residue, selenium yeast, oxidative stress, inflammatory cytokines, broiler chicken

## Abstract

Copper (Cu) could be seriously hazardous when present at excessive levels, despite its vital contribution to various cellular processes. Selenium-enriched yeast (SeY) was reported to improve the health and metabolic status in broiler chicken. Hence, our study was endeavored to illustrate the mitigating efficacy of SeY on Cu-induced hepatic and renal damage. Cobb chicks aged 1 day were allocated into four experimental groups and offered a basal diet, SeY (0.5 mg/kg), CuSO_4_ (300 mg/kg), or SeY plus CuSO_4_ in their diets for 42 days. Our results revealed that SeY supplement antagonized significantly the Cu accumulation in livers and kidneys of exposed birds. Marked declines were also detected in the AST, ALT, urea, and creatinine levels, besides marked increases in total protein, glycerides, and cholesterol in the SeY-supplemented group. Moreover, enhancement of cellular antioxidant biomarkers (superoxide dismutase, CAT, GPx, and GSH) along with lowered MDA contents were achieved by SeY in hepatic and renal tissues. Further, SeY exerted a noteworthy anti-inflammatory action as indicated by decreased inflammatory biomarkers (IL-1β and TNF-α) and NO levels in both organs. Noticeable histopathological alterations of both organs further validated the changes in the markers mentioned above. To sum up, our findings indicate that SeY can be considered a potential feed supplement for alleviating Cu-induced hepatic and renal damage in broilers, possibly *via* activation of antioxidant molecules and lessening the inflammatory stress.

## 1 Introduction

Heavy metals are naturally distributed inorganic compounds, that can be discharged from various sources and negatively affect the health of living organisms (Habotta et al., 2022). Among these hazardous environmental metals, copper (Cu) is an element of concern owing to its wide distribution and high toxicity (Zhao et al., 2018). Cu is widely incorporated in many agrochemicals as pesticides, including cupric sulfate (CuSO_4_), which is toxic to different living organisms. In addition, increased Cu release to the environment may happen through melting, mining, industrial, and waste removal activities (Yang et al., 2020). Since it is highly soluble in water, CuSO_4_ can easily disseminate to the environment; therefore, Cu exposure is inevitable to animals *via* polluted food or water (Liu et al., 2018a). Notably, it contributes substantially to cellular metabolism and numerous physiological processes such as hematopoiesis, mitochondrial respiration, antioxidation, and immunity (Yang et al., 2020). Despite its valuable physiological functions, former studies had reported that excess Cu could evoke toxicity and damage to hepatic, renal, nervous, and digestive systems (Hashem et al., 2021a; Liao et al., 2021). Cu can impair the respiratory enzyme complexes that stimulate the over-generation of highly reactive radicals and cellular oxidative injury (Hashem et al., 2021a).

The metabolism of Cu is controlled principally by the hepatic tissue, where it accumulates upon excess exposure with no noticeable signs. When the exposed Cu overwhelms the hepatic storage capability, hepatocellular lesions are developed together with release into the circulation causing damage to other tissues (Lu et al., 2010). Dietary exposure to CuSO4 elicited notable elevations in serum aminotransferases, alkaline phosphatase together with declines in total protein, albumin, globulins, triglycerides, total cholesterol, low-density lipoprotein-cholesterol, and high-density lipoprotein-cholesterol levels in broilers (Hashem et al., 2021b). Likewise, dietary supplementation of inorganic Cu at a dose of 150 mg/kg significantly decreased liver and meat lipids, cholesterol, plasma lipids, triglycerides and cholesterol, beside increasing plasma AST and ALT in exposed ducks (Attia et al., 2012). Further, Cu-intoxicated chickens had higher serum levels of urea, creatinine, and uric acid in comparison with the control group (Elazab et al., 2021). The deleterious effects of Cu are associated with reactive oxygen species (ROS) formation that surpasses the antioxidant defense system (Liu et al., 2018a). The cellular oxidative stress is accompanied by augmented inflammatory reaction by triggering the proinflammatory mediators (Liu et al., 2018b; Abdeen et al., 2021; Ismail et al., 2022). Liu et al. showed that dietary Cu exposure encouraged oxidative damage and peroxidation of lipid in chicken liver (Liu et al., 2018a). Notable suppression was observed in the antioxidant enzymes with triggered inflammatory responses in immune organs (Yang et al., 2020) and kidneys (Wang et al., 2017) of chicken fed with a Cu-contaminated diet. Further, Cu-induced oxidative damage was reported to trigger mitochondrial fragmentation leading to leakage of cytochrome-c, which in turn facilitates the cell death (Zhao et al., 2018).

Selenium is another essential element crucial for maintaining the intracellular redox balance *via* scavenging the harmful reactive radicals, thus alleviating cellular oxidative injury (Li et al., 2022). Selenium-enriched yeast (SeY) is an organic form of selenium that is lower in toxicity with higher digestibility and bioavailability than sodium selenide (Arnaut et al., 2021). Yeast cells can bind with selenium’s organic and inorganic forms and incorporate them permanently into their cell structure. Yeast can bioaccumulate and convert inorganic selenium (sodium selenate and sodium selenite) into organic forms (SeY) (Kieliszek et al., 2015). Supplementation of *Oreochromis niloticus* for SeY over a period of 60 days counteracted hypoproteinemia, elevated serum aminotransferases, urea, and creatinine induced by organophosphorus intoxication (Hassan et al., 2022). Se-enriched yeast reduced creatinine, and blood urea nitrogen levels in the kidneys of chromium-exposed broilers (Zhao et al., 2022). SeY was reported to counteract ochratoxin A-mediated suppression in the levels of antioxidant enzymes and genes in the hepatic and renal tissues of treated chickens (Li et al., 2020b; Li et al., 2020a). Former results unveiled that SeY protected against hepatic and renal oxidative stress and necroptosis elicited by cadmium toxicity in chicken (Wang et al., 2020a; Chen et al., 2021). Furthermore, SeY exerted a remarkable anti-inflammatory effect induced by lead by downregulating the gene transcription levels of inflammatory mediators in skeletal muscles such as interleukin (IL)-1β, IL-4, and IL-10 of exposed chicken (Liu et al., 2019). Similarly, dietary supplementation of chicken by 0.5 mg/kg SeY lessened markedly the expression levels of gene and protein related to hepatic inflammatory markers like iNOS, NF-κB, TNF-α, and prostaglandins (Wang et al., 2020b).

Up to our knowledge, the mitigating efficiency of SeY against Cu-evoked organ injury in broiler chickens has not been investigated. Therefore, this study was designed to explore the potential protective action of SeY as a feed supplement against Cu-induce oxidative and inflammatory stresses in liver and kidney tissues. Hence, this study appraises the protective impact of SeY against excess dietary inclusion of Cu and enriches its application for improvement of health status in young broiler chicks.

## 2 Materials and methods

### 2.1 Materials

Copper (II) sulphate pentahydrate (CuSO_4_.5H_2_O; CAS number 7758–99-8) was purchased from Sigma Aldrich Co. (St. Louis, MO, United States). Selenium yeast (Se, 2000 ppm) was provided by Angel Yeast (Hubei, China; purity >99:5%). All other used chemicals and reagents were of high analytical grade.

### 2.2 Experimental birds and protocol

One day old chicks of Cobb strain broilers were attained from the Faculty of Agriculture, Mansoura University, Egypt. They were raised in clean, well-ventilated floor pens under complete hygienic measures, one replicate per each pen (three birds/replication). Chicks were offered a balanced commercial ration and tap water *ad libitum*. The ideal temperature was changed; for example, it was set at 32°C for the first week and then dropped by 1°C each following week to reach 25°C. The lighting system was 24 h per day for the first week, then changed to 16 h of light and 8 h of darkness starting on day 7 and lasting until the completion of the experiment. The relative humidity was held between 60 and 70%. The basic diet was developed in accordance with Cobb 500 broilers’ Broiler Performance and Nutrition Supplement. From day 1 through day 10, birds were given a beginning diet; from day 11 through day 22 they were given a growing diet; from day 23 to the completion of the experiment, they were given a finisher diet. At 7 and 14 days old, all birds received vaccinations against Newcastle disease; at 11 and 22 days old, they received vaccinations against Gumboro disease (Giambrone and Clay, 1986). Table 1 lists the feed components and chemical makeup of the basic diet. The experiment design, procedures and techniques were done in accordance with the guidelines of the Institutional Animal Care and Use Committee of Faculty of Veterinary Medicine, Mansoura University (R/133).

**TABLE 1:** Proximate and chemical composition of the basal diets (%).

	Experimental diets		
	Starter	Grower	Finisher
**Ingredients (%)**			
Corn, yellow	59.39	63.41	69.17
Soybean meal 48% crude protein	30.00	26.83	18.92
Corn gluten 60% crude protein	4.53	3	6.30
Soybean oil	2.60	3.4	2.60
Lime stone	1.90	1.83	1.74
Dicalcium phosphate	0.41	0.33	0.20
Common salts	0.30	0.3	0.30
Vit. Premix^*^	0.25	0.25	0.25
DL Lysine HCL	0.39	0.35	0.38
DL methionine	0.13	0.15	0.08
L-Threonine	0.08	0.08	0.06
L-Valine	0.02	0.02	0
**Chemical composition (%)**			
Calculated crude protein	21.50	19.50	18.50
Calculated metabolized energy (Kcal/kg)	3034	3107	3180
Analyzed crude protein^*^	21.32	19.40	18.41
Analyzed ether extract*	5.40	5.82	5.80
Analyzed Ash	5.97	6.36	5.00
Calcium	0.9	0.84	0.76
Available Available phosphate	0.46	0.43	0.38

*Vitamins and minerals premix used to cover the required vitamins and minerals per each kilogram diet (Vit. A, 10,000 I.U.; Vit. D3, 1,500 I.U.; Vit. E, 10 mg; Vit. K3, 2 mg; Vit. B1, 2 mg; Vit. B2, 5 mg; Vit. B6, 3 mg; Vit. B12, 0.01 mg; Niacin, 27 mg; Folic acid, 1 mg; Biotin, 0.05 mg; Pantothenic acid, 10 mg; Mn, 60 mg; Zn, 50 mg; Cu, 10 mg; I, 0.1 mg; Se, 0.1 mg; Co, 0.1 mg; Fe, 50 mg).

### 2.3 Experimental protocol and sampling

The chicks were haphazardly allocated into four groups of 15 each, with 5 replicates in each group (3 birds x 5 replicates), as follows;

1 The first group (Con) received a daily, additive-free basal diet.
2 The second group (Cu) was dietary administered with 300 mg/kg CuSO4 following the method of Cinar et al. (Cinar et al., 2014; Hashem et al., 2021b).
3 The third group (selenium yeast; SeY) received a SeY-supplemented diet at a dose of 0.5 mg/kg (Wang et al., 2020a; Chen et al., 2021).
4 The last group (Cu+SeY) was administered basal diets supplemented with CuSO_4_/kg diet plus SeY at previously mentioned doses.

All birds were carefully observed for any abnormal signs during the experimental time, which lasted for 42 days. Six randomly chosen birds from each group had blood drawn from their wing veins, which were then centrifuged at 3000 x g for 10 min. Sera were then separated and kept at -20°C in deep freezing until further biochemical analyses. Following the recommendations of the American Veterinary Medical Association (Schaumburg, IL, United States), the birds were killed *via* cervical dislocation (Leary et al., 2016). Liver and kidney tissue were divided into different portions to evaluate antioxidant enzymes, inflammatory biomarkers, Cu and Se residues in both tissues, and histological alterations.

### 2.4 Assessment of Cu and Se concentrations in liver and kidney

Following the mineral measurement method (AOAC) (Chemists and Chemists, 1925), 3 ml of concentrated nitric acid and 1.5 ml of concentrated perchloric acid were used to digest 0.5 gm of liver or kidney tissue. The digestion process was then finished by incubating the samples in a water bath adjusted at 53°C/overnight. The resulting mixture was filtered, left to cool to room temperature, and then 20 ml of deionized water was added for dilution. The concentrations of Cu and Se were determined using a flame atomic absorption spectrophotometer (Buck Scientific 210 VGP, Inc., Norwalk, Connecticut, CT, United States).

### 2.5 Hepatic function parameters

As directed by the manufacturer, serum samples were utilized to calculate hepatic and renal damage indications. According to Reitman and Frankel, the enzymatic activity of alanine aminotransferase (ALT) and aspartate aminotransferase (AST) were estimated (Reitman and Frankel, 1957). Following the protocol of Lowry et al., the serum level of total protein was examined (Lowry et al., 1951). In addition, Roeschlau et al. (1974) and McGowan et al. (1983) were used to estimate the total serum cholesterol and triglycerides levels, respectively.

### 2.6 Renal function biomarkers

According to Coulombe and Favreau’s (Coulombe and Favreau, 1963) and Larsen’s (Larsen, 1972), we evaluated the renal function markers, urea and creatinine.

### 2.7 Hepatorenal oxidative stress markers

The peroxidation of lipids was assessed spectrophotometrically by measurement of its secondary metabolite, malondialdehyde (MDA), in liver and kidney homogenates, according to Ohkawa et al. (1979). Depending on the reducing ability of glutathione to 5,5′-dithiobis (2-nitrobenzoic acid) to give yellow-colored 5-thionitrobenzoic acid, glutathione (GSH) was analyzed spectrophotometrically at 405 nm as stated by Ellman (1959).

### 2.8 Hepatorenal antioxidant enzymes activities

The activity of superoxide dismutase (SOD) was evaluated depending on the nitroblue tetrazolium dye reduction rate to diformazan following the method established by Sun et al. (1988). Catalase (CAT) activity was assessed based on the depletion of H_2_O_2_ into H_2_O and molecular O at 240 nm, as mentioned by Aebi (1984). Additionally, the evaluation of glutathione peroxidase (GPx) activity was assessed by Paglia and Valentine (1967).

### 2.9 Hepatorenal inflammatory biomarkers

Griess reagent was utilized to estimate nitrite/nitrate (Nitric oxide; NO) levels as stated by Green et al. (1982). The concentrations of IL-1β (Cat number MBS261118) and TNF-α (Cat number MBS2509660) were analyzed using specific ELISA assay kits (MyBioSource, CA, United States).

### 2.10 Histopathological investigations

Liver and kidney specimens were fixed in 10% neutral buffered formalin for 24 h, dehydrated, cleared with xylene, and embedded in molten paraplast. For microscopical examination, a 5 µm thick paraffin section was stained with hematoxylin and eosin.

### 2.11 Statistical analyses

Obtained data were analyzed by one-way analysis of variance (ANOVA) followed by *post hoc* Duncan’s multiple range test to decide the significance among groups. Data were displayed as mean ± standard error (SE). At *p* values <0.05, statistical significance was determined between groups. A multivariate analysis among variables and different treatments was performed through the principal component analysis (PCA) conduction. Together, a clustering heatmap was analyzed by RStudio (R version 4.0.2).

## 3 Results

### 3.1 Selenium yeast decreased hepatorenal Cu bioaccumulation

**FIGURE 1:**
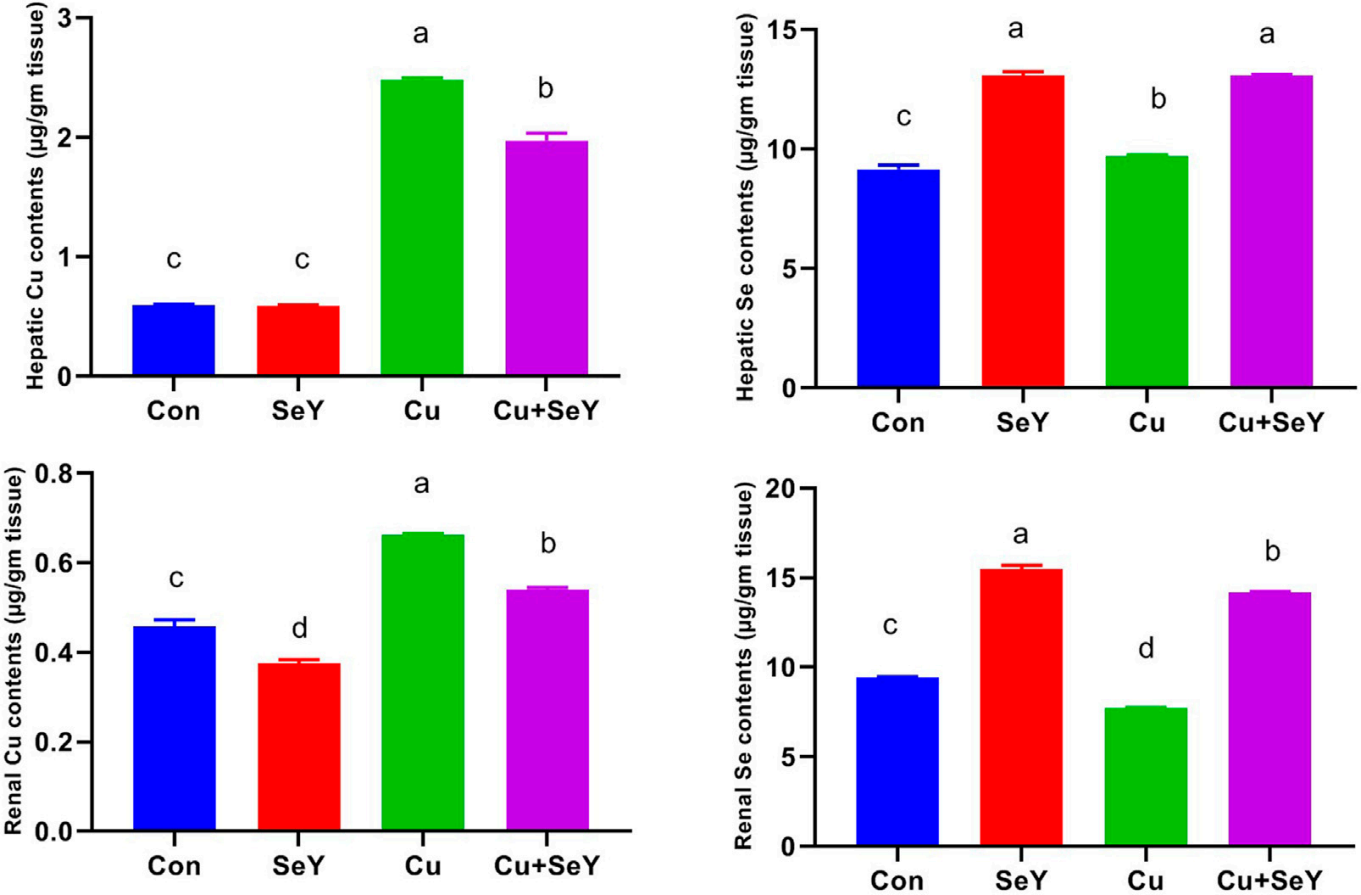
The copper (Cu) and selenium (Se) residual levels in the hepatic and renal tissues following dietary exposure to copper sulphate (CuSO_4_, 300 mg/kg) and/or selenium yeast (SeY, 0.5 mg/kg) in broiler chicken for 42 days. The values were represented as means ± SE (n = 6). Each bar carrying different letters is significantly different (*p* < 0.05).

In relation with the control group, significant increases (*p* < 0.05) were seen in the Cu contents in the liver and kidney of chickens that received dietary CuSO_4_, as illustrated in Figure 1. Contrarily, the Cu level in the Cu+SeY group was markedly less than (*p* < 0.05) in the Cu-intoxicated group. Moreover, there was no discernible difference in hepatic Cu levels between the control and SeY groups. SeY group’s renal Cu levels were lower than those of the control group.

Additionally, measurements of the Se content in both organs were made across all groups. The Se contents significantly increased (*p* < 0.05) in the Cu+SeY and SeY administered groups but notably diminished (*p* < 0.05) in the Cu-exposed chickens (Figure 1).

### 3.2 Selenium yeast modulated the serum biochemical markers

**FIGURE 2:**
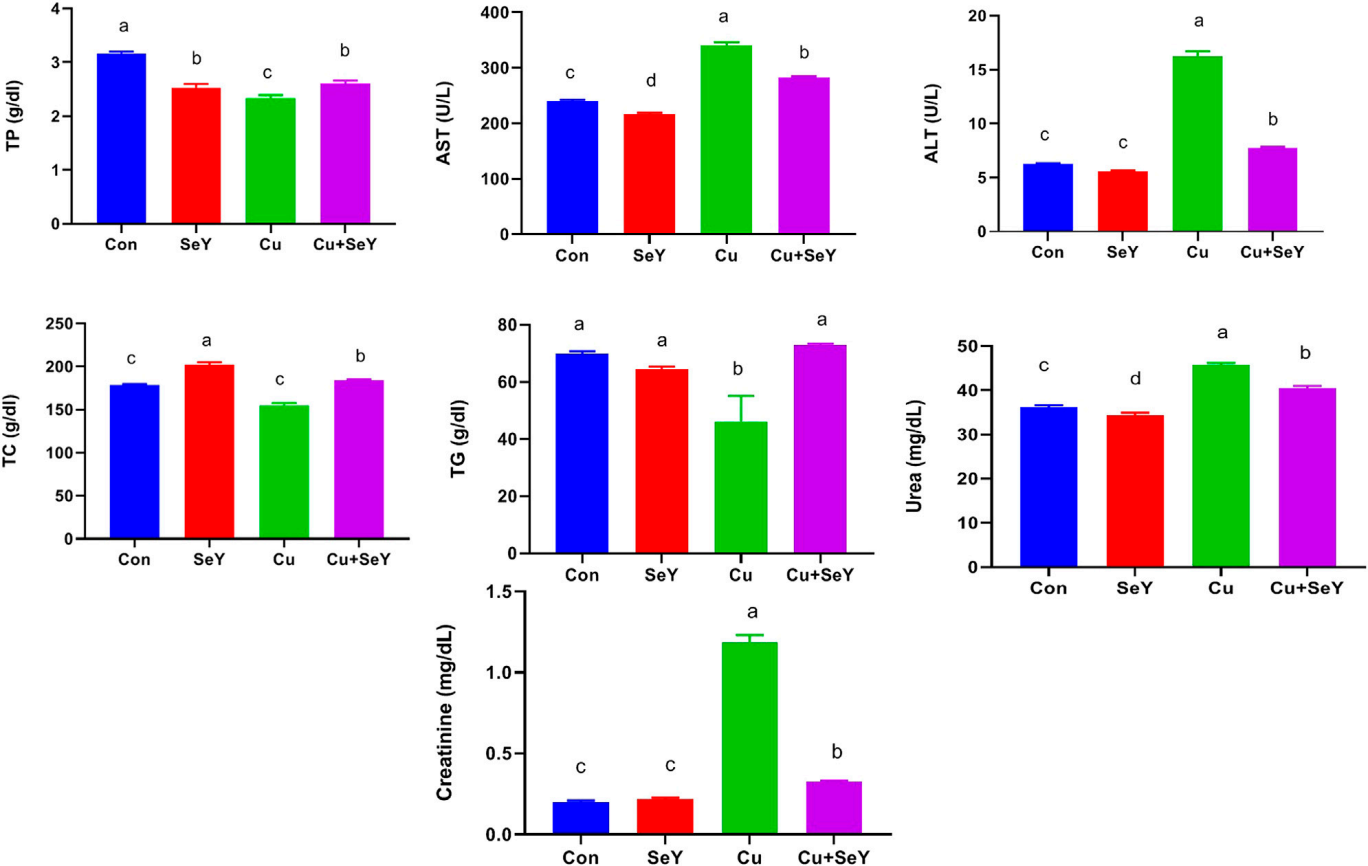
Serum biochemical markers following dietary exposure to copper sulphate (CuSO_4_, 300 mg/kg) and/or selenium yeast (SeY, 0.5 mg/kg) in broiler chicken for 42 days. The values were represented as means ± SE (*n* = 6). Each bar carrying different letters is significantly different (*p* < 0.05).

The indices of liver integrity, including TP, ALT, and AST were measured in serum samples. Noteworthy rises (*p* < 0.05) were noticed in ALT and AST activities with marked decreases in TP in respect to the control group. Adversely, dietary supplementation with SeY markedly reversed (*p* < 0.05) Cu-induced alterations relative to the Cu-treated group (Figure 2). In addition, marked declines (*p* < 0.05) were noticed in serum levels of TC and TG in the Cu-treated group in relation to the sham group, but the supplement of SeY resulted in prominent rises (*p* < 0.05) in their level compared to Cu treated only (Figure 2).

Regarding the effect of SeY on Cu-induced renal functions, serum levels of urea and creatinine were investigated. Our results showed that dietary Cu exposure induced substantial increments (*p* < 0.05) in urea and creatinine levels in relation to the control. However, their levels notably decreased (*p* < 0.05) with the combined treatment with Cu plus SeY (Figure 2).

### 3.3 Selenium yeast decreased Cu-mediated hepatorenal oxidative stress

**FIGURE 3:**
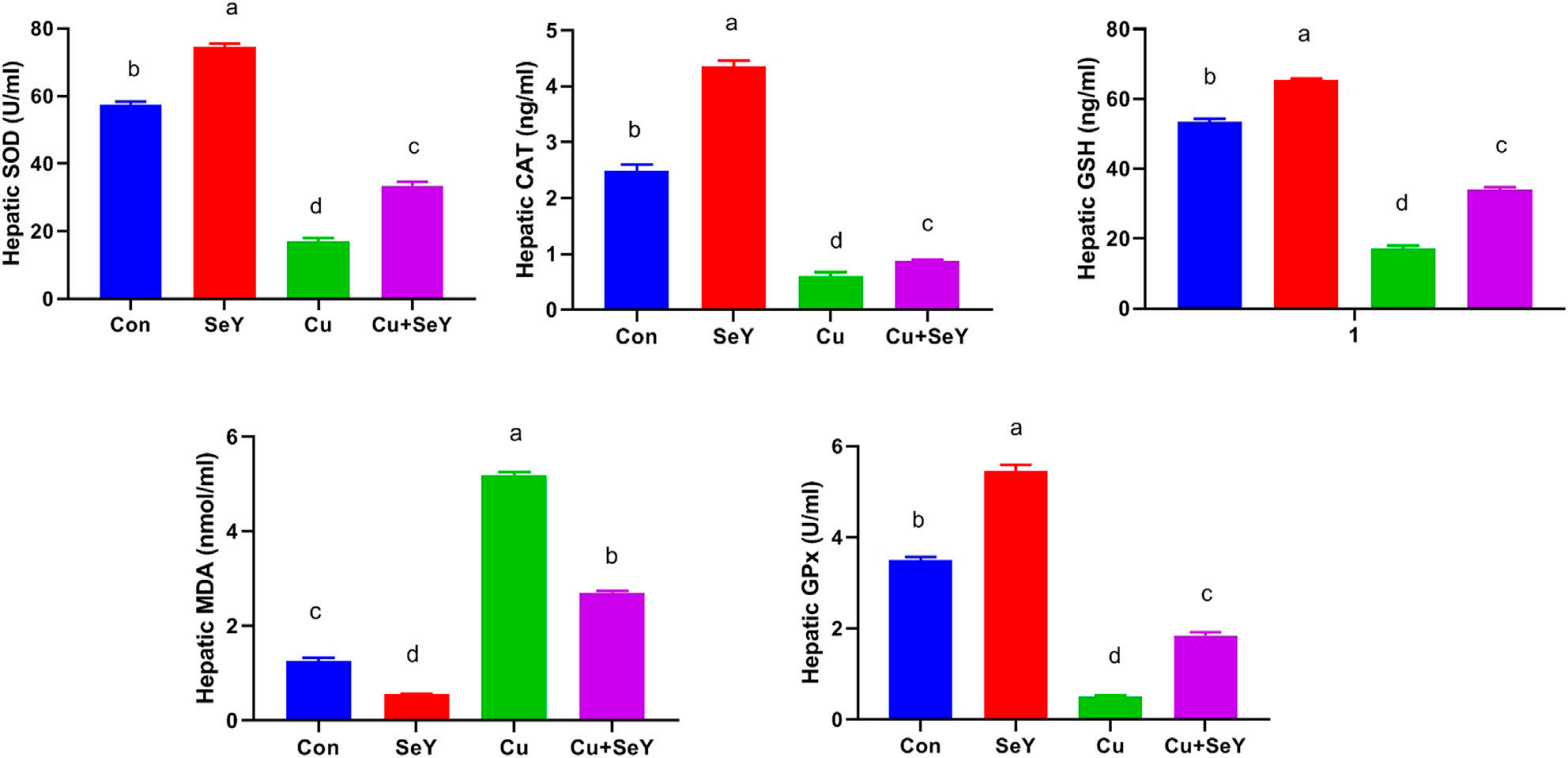
Changes in the glutathione peroxidase (GPx), superoxide dismutase (SOD), and catalase (CAT) activities and reduced-glutathione (GSH) and malondaialdehyde (MDA) levels in the liver tissue following dietary exposure to copper sulphate (CuSO_4_, 300 mg/kg) and/or selenium yeast (SeY, 0.5 mg/kg) in broiler chicken for 42 days. The values were represented as means ± SE (n = 6). Each bar carrying different letters is significantly different (*p* < 0.05).

To investigate the impact of Cu and/or SeY on hepatic oxidative stress, enzymatic and non-enzymatic biomarkers were investigated. As shown in Figure 5, significant depletions were noticed in the contents of SOD, CAT, GPx, and GSH (*p* < 0.05) in the Cu-administered group compared to the control group. These changes were obviously reversed (*p* < 0.05) after dietary supplementation with SeY. The lipid peroxidation expressed in MDA level showed a substantial rise (*p* < 0.05) in hepatic tissue of Cu group in relation to the control. The SeY group had a marked lower MDA level (*p* < 0.05) than that of the group that received Cu only (Figure 3).

**FIGURE 4:**
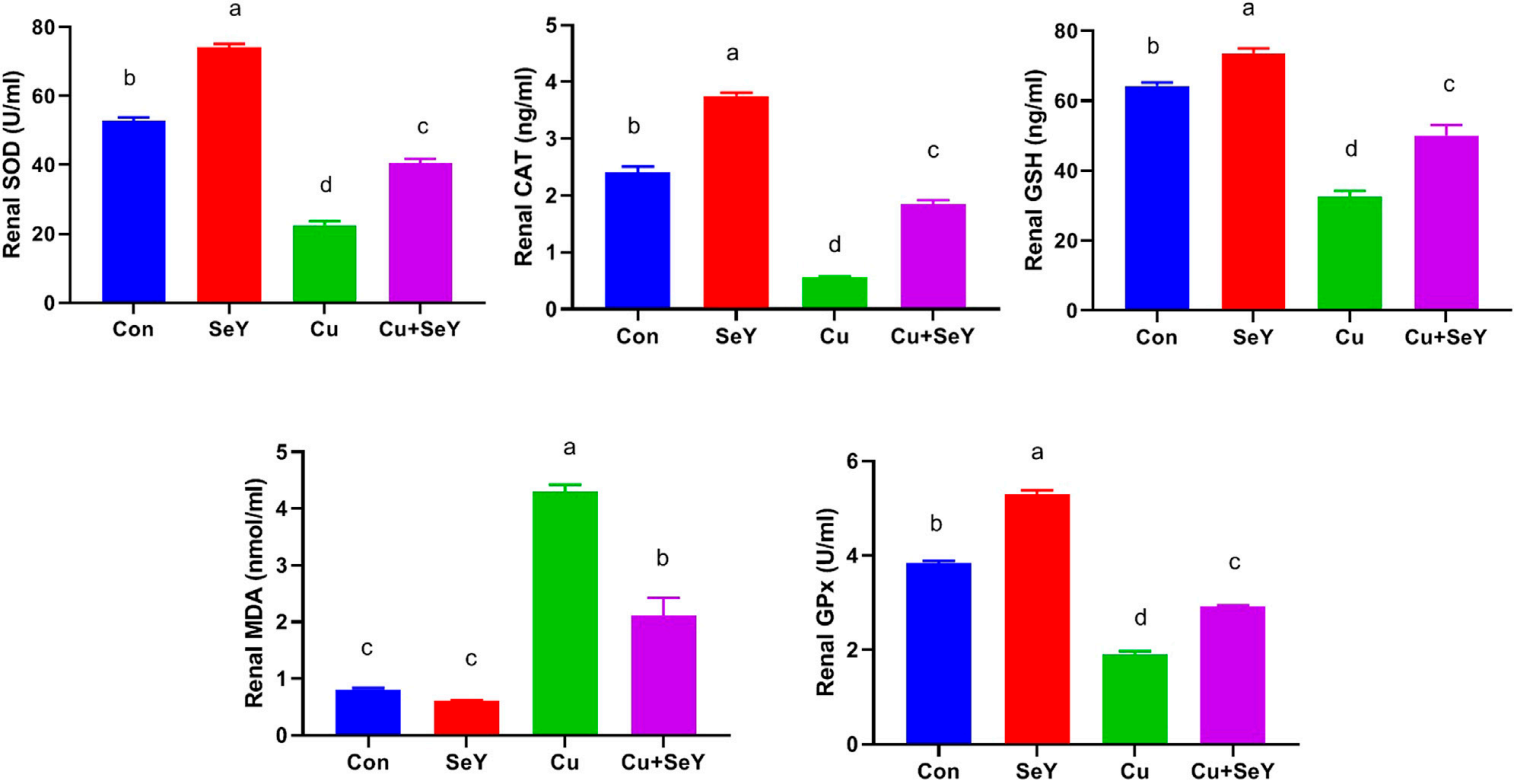
Changes in the glutathione peroxidase (GPx), superoxide dismutase (SOD), and catalase (CAT) activities and reduced-glutathione (GSH), and malondaialdehyde (MDA) levels in the renal tissue following dietary exposure to copper sulphate (CuSO_4_, 300 mg/kg) and/or selenium yeast (SeY, 0.5 mg/kg) in broiler chicken for 42 days. The values were represented as means ± SE (n = 6). Each bar carrying different letters is significantly different (*p* < 0.05).

The renal contents of SOD, CAT, GPx, and GSH were meaningfully decreased (*p* < 0.05) in CuSO_4_-exposed chicken compared to the controls. Contrarily, a remarkable decline (*p* < 0.05) was detected in renal MDA level in CuSO_4_ group relative to the sham group. Moreover, the Cu-induced alterations in renal lipid peroxide level and antioxidative biomarkers were counteracted by SeY supplementation compared to the Cu group (Figure 4).

### 3.4 Selenium yeast blocked Cu-mediated hepatorenal inflammation

**FIGURE 5:**
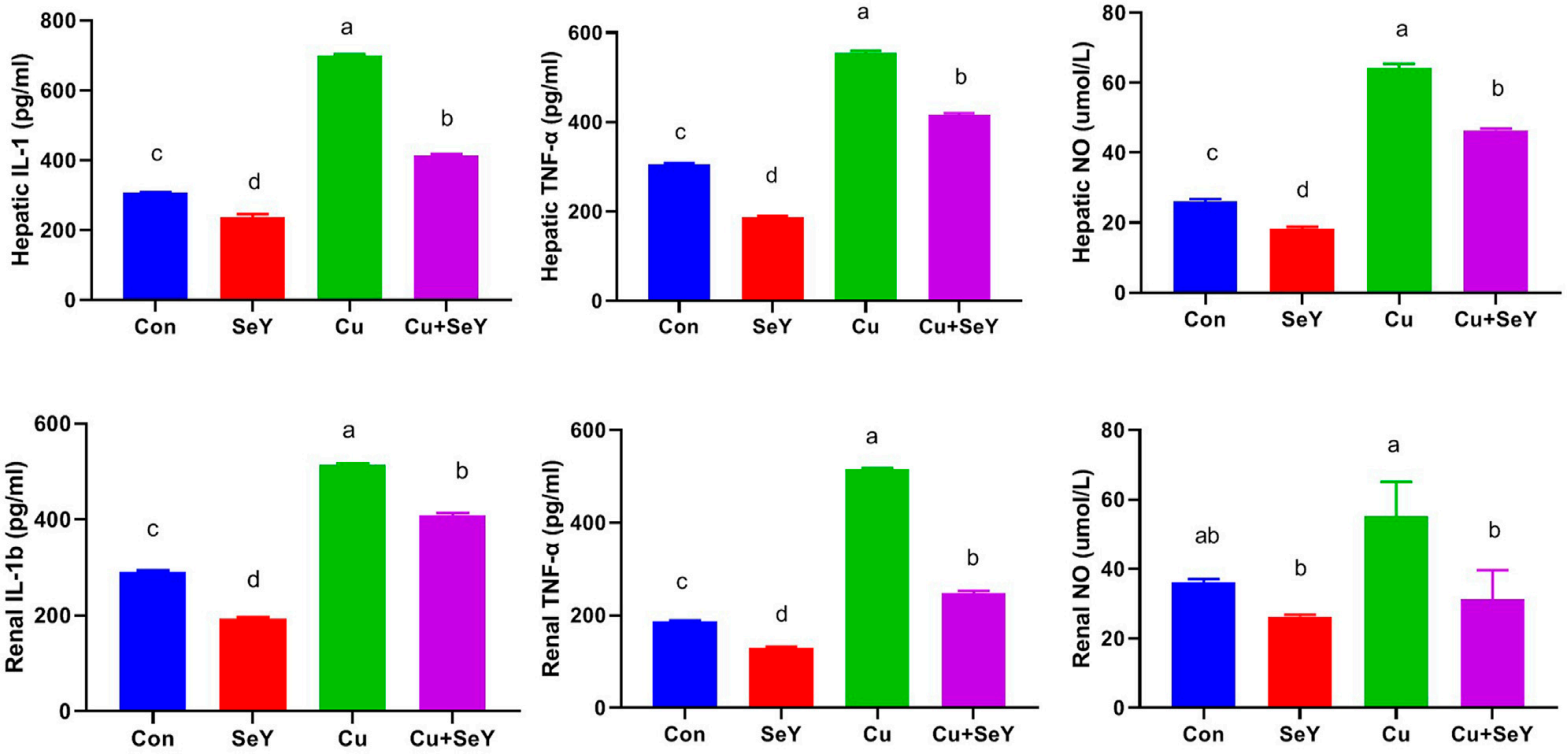
Hepatic and renal levels of IL-1β, TNF- α, and NO following dietary exposure to copper sulphate (CuSO_4_, 300 mg/kg) and/or selenium yeast (SeY, 0.5 mg/kg) in broiler chicken for 42 days. The values were represented as means ± SE (*n* = 6). Each bar carrying different letters is significantly different (*p* < 0.05).

As depicted in Figure 5, the CuSO_4_-exposed group displayed meaningfully higher levels (*p* < 0.05) of pro-inflammatory cytokines, such as IL-1β and TNF-α, along with NO, in both tissues compared to controls. However, after SeY supplementation, their levels declined (*p* < 0.05) compared to the Cu-treated group, indicating the anti-inflammatory potential of SeY dietary inclusion.

### 3.5 Selenium yeast alleviated hepatorenal pathological alterations evoked by Cu

**FIGURE 6:**
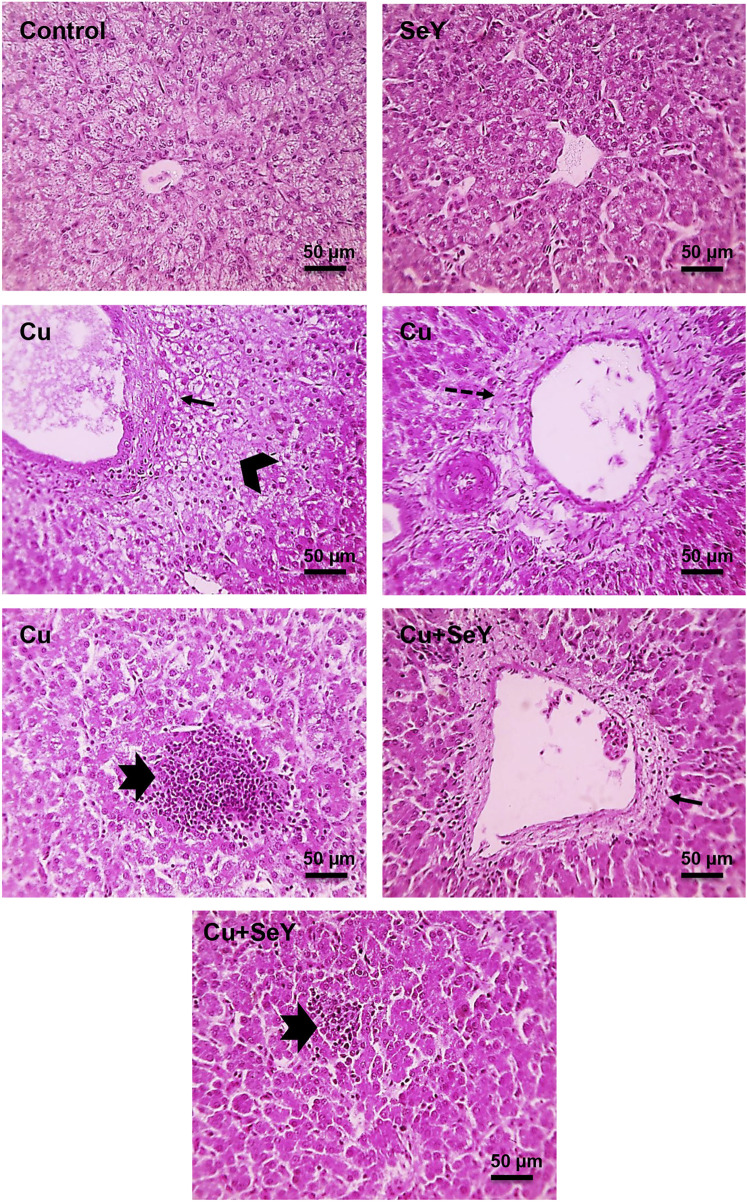
Microscopic picture of HE-stained liver sections from Control and SeY groups show normally arranged hepatocytes in radial plates around central veins with normal sinusoids and portal areas. However, liver sections from Cu group show portal fibrosis (thin black arrows), periportal coagulative necrosis of hepatocytes (black arrowheads), large lymphocytes follicular aggregation (thick black arrows). Cu+SeY group exhibited mild portal fibrosis (thin black arrows) with small lymphocytes follicular aggregation (thick black arrows). Bars = 50 µm.

Microscopic pictures of hepatic sections from a control group and group that received SeY showed normally arranged hepatocytes in radial plates around central veins with normal sinusoids and portal areas. Further, liver sections in birds received Cu showing portal fibrosis, periportal coagulative necrosis of hepatocytes, and large lymphocyte follicular aggregation. In contrast, mild portal fibrosis with small lymphocytes follicular aggregation was seen in Cu+SeY group (Figure 6).

**FIGURE 7:**
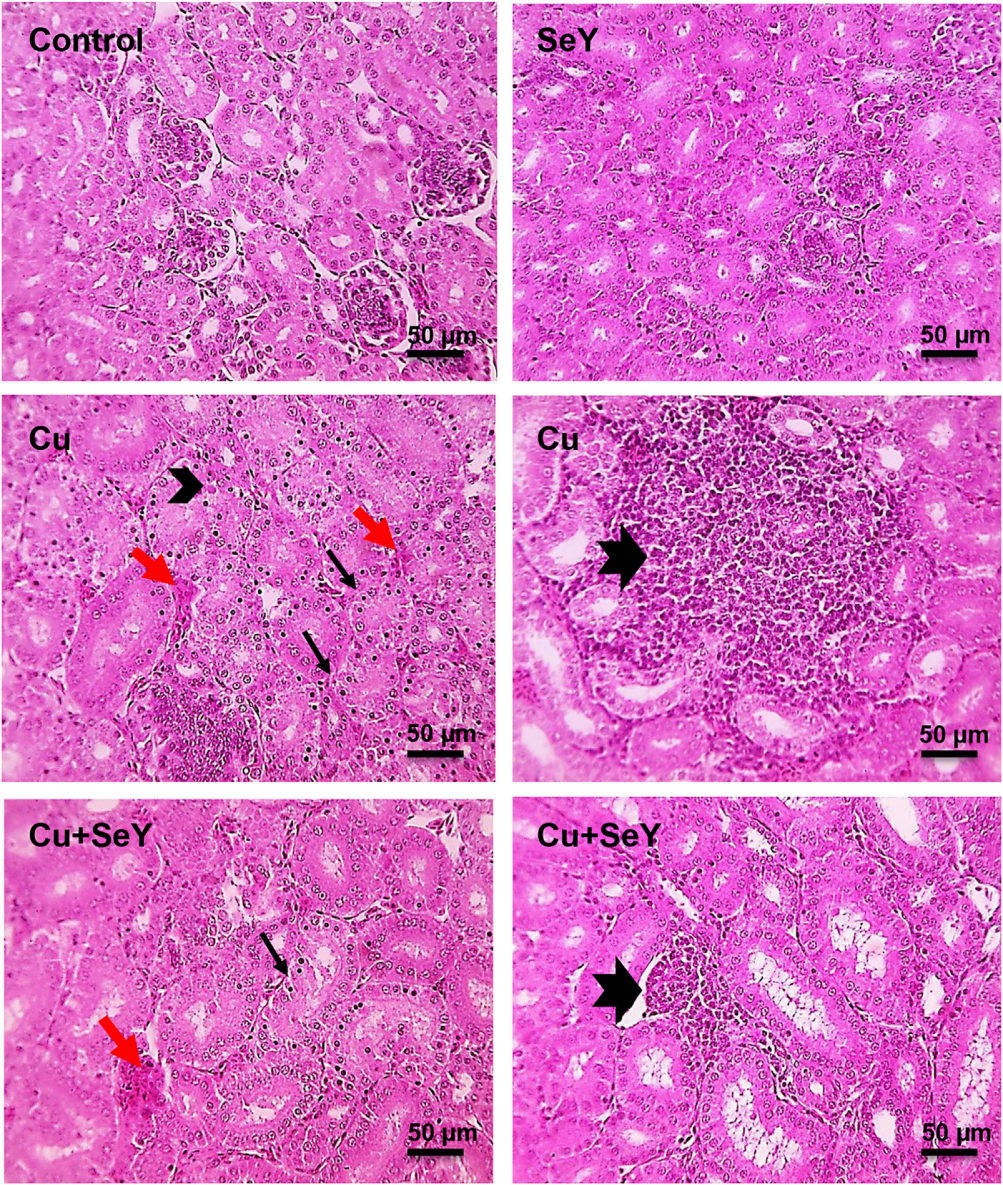
Microscopic pictures of HE-stained kidney sections from Control group and SeY group show normal tubules, glomeruli, and interstitial tissue. Kidney sections from Cu-treated group exhibit prominent tubular necrosis (thin black arrows), few apoptotic cells (arrowheads), congested inter-tubular blood vessels (red arrows), and large lymphocytes follicular aggregation in interstitial tissue (thick black arrows). Cu+SeY group presents mild tubular necrosis (thin black arrows), congested inter-tubular blood vessels (red arrows), and small lymphocytes follicular aggregation in interstitial tissue (thick black arrows). Bars = 50 µm.

Microscopical screening of kidney sections from control and SeY groups showing normal tubules, glomeruli, and interstitial tissue. However, those from Cu-intoxicated birds exhibited prominent tubular necrosis, few apoptotic cells, congested inter-tubular blood vessels, and severe lymphocytic infiltration. On another hand, mild tubular necrosis, congested inter-tubular blood vessels, and mild lymphocytic aggregation in interstitial tissue were recorded after supplementation of SeY to the Cu-exposed chicks (Figure 7).

### 3.6 Variable influence and clustering heatmap

**FIGURE 8:**
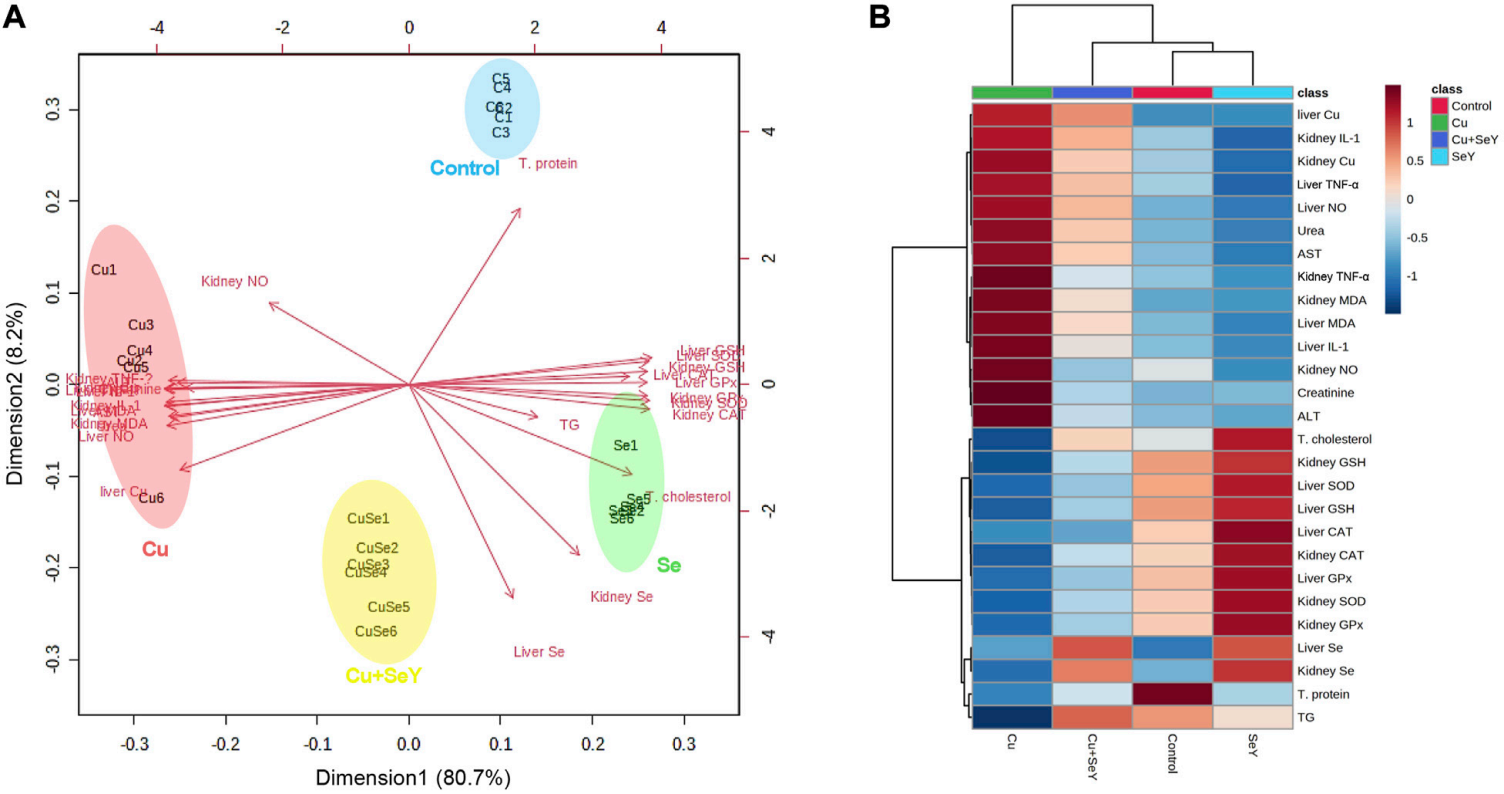
Principal components analysis (PCA) and clustering heatmap of SeY versus Cu-induced hepatorenal damage. **(A)** Score biplot of PCA and variable contribution. **(B)** Clustering heatmap enables intuitive visualization of all variable concentration values (dark red is the highest and blue represents the lowest values). C1-6, Samples of Control group; CAT, catalase; Cu1-6, Samples of Cu group; CuSe1-6, Samples of Cu+SeY group; GPx, glutathione peroxidase; GSH, reduced-glutathione; MDA, malondialdehyde; Se1-6, Samples of SeY group; SOD, superoxide dismutase; TG, triglycerides; T. protein, total protein.

A multivariate analysis (principal component analysis, PCA) was conducted and a smaller set of “summary indices” were elaborated as exhibited in Figure 8A. These data set showed how the studied variables contributed and grouped together along Dimension1 and 2 in response to different treatments. The present PCA indicated that Dimension 1 and 2 had the major contribution (80.7% and 8.2%, respectively). In the same data frame, the PCA score plot revealed that NO, TNF-α, IL-1β, MDA, urea, creatinine, and Cu residues have a tendency to change in the same way in response to Cu toxicity, hence they are grouped oppositely to the Control and SeY groups. Interestingly, the Cu+SeY group is located in the middle between both arms.

Moreover, the clustering heatmap seen in Figure 8B summarizes the concentrations of all determined variables in groups. The heatmap shows the variable concentrations in the Cu-exposed birds are negatively correlated to the same corresponding concentrations in other treatments.

## 4 Discussion

The current experiment unveiled that SeY was able to reverse the hepatorenal impairment raised by Cu toxicity *via* improving organ functions, enhancing the antioxidant enzymatic activity, and lessening the tissue inflammation in growing broiler chicks. The excess Cu which cannot be digested and absorbed can induce liver injury and negatively affects other organs through circulation (Gaetke et al., 2014). In our study, we measured both Cu and Se levels in hepatic and renal tissues. The results revealed that chicken feeding on a Cu-containing diet resulted in increases in its residues in the liver and kidney compared to the control birds. Former studies showed that hepatic Cu retention increased with dietary Cu supplementation (Kim and Kil, 2015; Wu et al., 2020). Another study employed in chickens indicated that the amount of Cu in the kidney increased somewhat as the amount of time exposed to Cu increased (Elazab et al., 2021). Interestingly, the supplementation with SeY conferred a significant antagonistic action against hepatorenal Cu accumulation in chicken. Lui et al. observed that SeY supplementation at a dose of 0.3 mg/kg for 35 days could markedly decrease lead overload in the skeletal muscles of intoxicated chickens (Liu et al., 2019). Additionally, raising chicken on 3 mg/kg SeY for 90 days evoked a noticeable reduction in the accumulation of cadmium in the chicken heart (Ge et al., 2021a). These findings indicated that SeY possesses an efficient metal chelation power *via* forming inactive complexes with heavy metals and enhancing its excretion with subsequent decreases in their concentrations.

The accumulation of excess Cu in both hepatic and renal tissues resulted in disturbance in these organs’ functions. Our findings showed significant increases in serum transaminases and decreased serum TP levels in the Cu-exposed group, implying hepatotoxicity because the liver is the central location for Cu accumulation. These findings agree with former studies (Wu et al., 2020; Hashem et al., 2021a). This may be endorsed for the hepatic damage that resulted in excess release of hepatic intracellular enzymes into the bloodstream (Atta et al., 2009). Additionally, the observed hypoproteinemia in our results may refer to the impairment in protein synthesis because of liver damage and/or excess protein loss due to renal insufficiency (Al Aboud et al., 2021). These results are well reinforced by the histopathological findings’ periportal coagulative necrosis of hepatocytes. However, supplementation of 450 mg/kg copper proteinate decreased ALT in Bovans laying hens after exposure for 4 weeks, that may be due to the slightly restricted food consumption (Güçlü et al., 2008).

On the contrary, the present study showed that the dietary supplement SeY improved these liver damage biomarkers and decreased the hepatic histological irregularities caused by Cu stress. Similar outcomes were stated by Malyar et al, (2021), who found that selenium-rich *Saccharomyces cerevisiae* restored the liver function biomarkers in rats kept under high heat stress for 42 days. Moreover, SeY protected against ochratoxin-induced elevations in hepatic AST and ALT in chicken exposed to a contaminated diet for 21 days (Li et al., 2020b). Hence, the modulating effect of SeY on liver function markers indicated its protective effect on the cell membrane of hepatocytes and block the enzyme leak into the blood circulation.

Since the kidney is the leading platform for the excretion of Cu, the renal tubules are vulnerable to Cu harm (Elazab et al., 2021). The analysis of renal function tests unveiled momentous upsurges in serum levels of urea and creatinine in CuSO_4_-intoxicated birds. As the kidney discharges the nitrogenous end products of the catabolic process, the increases in both biomarkers indicate the impairment in kidney functions (Abdeen et al., 2021; Othman et al., 2021). The histopathological screening of the kidney validated these results characterized by prominent tubular necrosis, few apoptotic cells, congested inter-tubular blood vessels, and large lymphocytes follicular aggregation in the interstitial tissue. These outcomes align with former reports illustrating the adverse renal pathology induced by Cu toxicity in various animal models (Baruah et al., 2018, Hashem et al., 2021b). Wang and colleagues (Wang et al., 2017) reported that chickens exposed to CuSO_4_ at 300 mg/kg food level for 12 weeks also developed atrophied glomeruli and tubular casts. The tubular cells also experienced degeneration and necrosis. Hence, it could be concluded that Cu impaired both glomerular and tubular functions with deteriorations of overall renal performance. Remarkably, this study’s findings showed that SeY obviously counteracted Cu-encouraged renal dysfunction in exposed chicken. Ge et al, (2021b) reported similar results in cadmium-exposed chicken and co-treated with SeY for 90 days. Also, renal function-related biomarkers (creatinine, urea, and uric acid) displayed significant decreases in the SeY-supplemented chicken related to the ochratoxin-intoxicated group (Li et al., 2020a). This nephroprotective effect of SeY refers to its antioxidant effect, and this was confirmed pathologically by the improved renal histoarchitecture.

Our results also showed that birds supplemented with excess CuSO_4_ had marked declines in serum TG and TC compared with the controls, which coincides with previous reports (Idowu et al., 2011; Hashem et al., 2021a). The rate-limiting enzyme in the catabolic process of cholesterol 7-hydroxylase was found to be more active when Cu-supplemented meals were administered (Konjufca et al., 1997). Further, adding Cu to the chicken diet diminished the contents of GSH which suppressed the activity of β-methylglutaryl-CoA reductase with a subsequent decrease in the cholesterol level (Kim et al., 1992). Additionally, dietary Cu caused substantial drops in the hepatic lipogenic enzyme activity, 17 beta-estradiol, and plasma lipid levels (Pearce et al., 1983). Therefore, these reductions in TC and TG of Cu-exposed chickens are caused by reduced cholesterol synthesis, increased lipid degradation, or excretion rate. However, different outcomes were reported by earlier studies. Cinar and collaborators did not find any alteration in plasma total cholesterol levels in copper-exposed broilers (Cinar et al., 2014). Additionally, marked decreases were reported in plasma triglycerides and cholesterol in Arbor-Acre unsexed broilers exposed to dietary CuSO_4_ or copper proteinate at 50, 100, or 150 mg/kg doses for 56 days (Jegede et al., 2011). These differences may endorse breed, diet components, and the investigational strategy. On the other side, the administration of SeY decreased the opposing effect of Cu on lipid metabolism-related markers. This might be attributed to the capacity of SeY to scavenge free radicals and defense against lipid structure peroxidation.

In addition to the induction of hepatorenal impairments in exposed broilers, Cu elicits over-generation hydroxyl radicals and hydrogen peroxide *via* Fenton and Haber-Weiss reactions (Wang et al., 2018). Many scholars have pointed out that the excess generation of ROS that overwhelm the cellular capacity is one of the hallmarks of heavy metals’ harmful actions (Albarakati et al., 2020; Kassab et al., 2020; Al Aboud et al., 2021). These highly reactive radicals could modify the structure or/and function of cellular molecules. The current findings revealed that chickens exposed to dietary Cu developed an imbalance in their liver and kidney’s oxidant/antioxidant status. SOD can hamper the superoxide anion and convert it into H_2_O_2_, which CAT dissociates into water (Albarakati et al., 2020). GPx significantly contributes to the protective function of CAT and is required for the regeneration of GSH. The significant increase in the levels of MDA in both organs indicated that Cu exposure enhanced the formation of OH^•,^ which directly interacted with the polyunsaturated fatty acids in cellular membrane lipid, which led to lipid peroxidation (Wang et al., 2018). Zhao et al. (Zhao et al., 2018) found significant decreases in SOD activity and GSH content in the chicken jejunum in a time-dependent manner after dietary exposure to 300 mg/kg Cu. In another related study, decreases in SOD, CAT, and GPx with increases in MDA in the immune organs were reported in chicken fed on a diet containing Cu at different concentrations for 49 days (Yang et al., 2020). Nevertheless, CuSO4 exposure for 30 days did not evoke significant differences in total antioxidant capacity in chicken jejunum (Zhao et al., 2018).

In contrast, our data demonstrated that SeY could enhance the hepatorenal antioxidant capacity by decreasing MDA levels and restoring the activity of GPx, SOD, CAT, and GSH concentrations. Se is a crucial cofactor for enzymes involved in scavenging ROS and dietary supplements of sodium selenide, and SeY increases birds’ stress tolerance (Arnaut et al., 2021). Li et al. recorded upregulation of hepatic GPx, GLRX2, and MnSOD mRNA expression along with Nrf2 protein expression and its downstream (Keap-1 and HO-1) after dietary inclusion with 0.4 mg/kg SeY in broilers (Li et al., 2020a) suggesting the possible involvement of Nrf2/HO-1/Keap-1 in the protective role of SeY (Li et al., 2020b). Our data were in agreement with those obtained by Cao et al. who reported a reduced aluminum-induced testicular damage in mice after SeY supplementation *via* modifying the redox status (Cao et al., 2020). Elevated ROS can trigger the production of pro-inflammatory cytokines and different inflammatory cells and the release of inflammatory mediators (AL-Megrin et al., 2020). High dietary Cu exposure has been documented to enhance the IL-1β and TNF-α levels in chicken immune organs (Yang et al., 2020) and liver (Liu et al., 2018a). Concurrently, the existing experiment presented that Cu intoxication caused significant elevations in the IL-1β and TNF-α. Interestingly, the proposed findings indicated that SeY could antagonize the Cu-enhanced hepatic and renal inflammatory responses. Se-rich *S. cerevisiae* downregulated the hepatic gene expression of IL-6, TNF-α, COX-2, and NF-κB of heat stressed (Malyar et al., 2021) and aluminium-intoxicated (Luo et al., 2018) Wister rats. Supporting a former study (Cao et al., 2020), SeY significantly mitigated aluminum-induced testicular toxicity by decreasing the level of NO and NOS activities. Therefore, we strongly assume that SeY might mitigate hepatorenal inflammation induced by Cu exposure *via* down-regulation of the inflammatory mediators.

**FIGURE 9:**
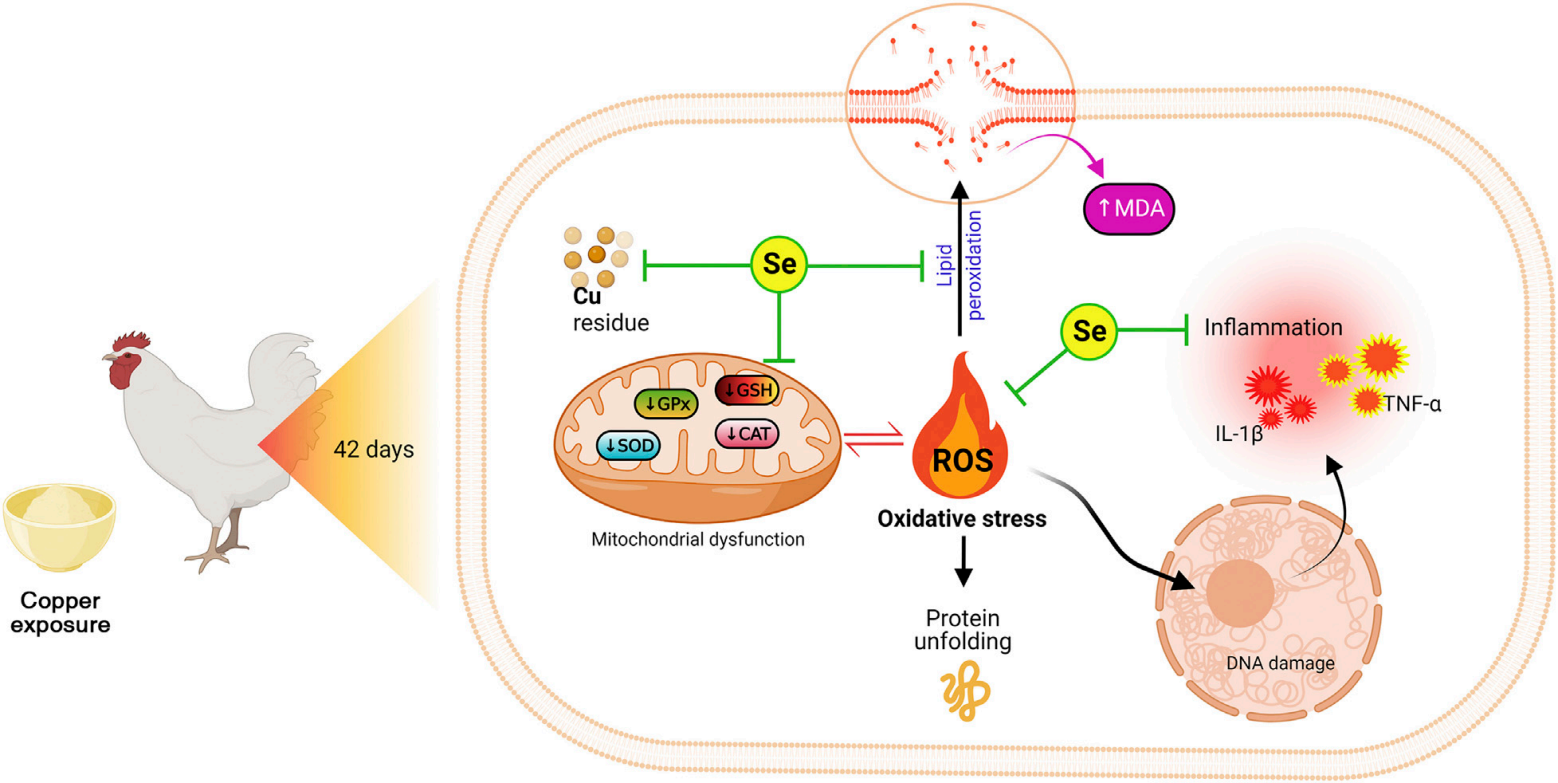
The molecular mechanisms located behind the ameliorative action of SeY toward Cu-stressed chickens.

Moreover, the PCA data indicated that all studied variables are clustered into four zones along Dimension1 (80.7% contribution) and Dimension2 (8.2% contribution). Such distribution was depending on different treatments, where, Cu-intoxicated birds were clustered on the left side and could be markedly discriminated from other treated groups confirming the occurrence of Cu toxicity. PCA also confirmed the protective effects of SeY supplementation since the Cu+SeY group has deviated to the midplane between Cu-intoxicated birds and non-intoxicated ones (Control and SeY groups). Moreover, the clustering heatmap summarizes the concentration levels of all measured parameters among different groups. The heatmap suggests that the variable concentrations in the Cu-intoxicated group are negatively correlated to the same corresponding concentrations in other groups. The molecular mechanisms located behind the ameliorative action of SeY toward Cu-stressed chickens are illustrated in Figure 9.

## 5 Conclusion

Collectively, this study introduced the alleviating effect of SeY against Cu-induced liver and kidney damage. SeY could reduce the extra-release of inflammatory mediators (IL-1β, TNF-α, and NO) and suppress lipid peroxidation and metal bioaccumulation. These protective mechanisms may be achieved by enhancing the antioxidant enzymatic of SOD, CAT, and GPx, alongside elevating the GSH contents. We strongly suggest that SeY could be a potential feed supplement that offers therapeutic evidence against the Cu-induced liver and kidney injury in chickens. In the future studies, further investigations are required for unveiling the underlying mechanisms for the antagonistic efficacy of Se-Y against heavy metal-induced damage in birds.

## Data Availability

The original contributions presented in the study are included in the article/supplementary material, further inquiries can be directed to the corresponding authors.
